# Brazil-Africa technical cooperation in health: what’s its relevance to the post-Busan debate on ‘aid effectiveness’?

**DOI:** 10.1186/1744-8603-9-2

**Published:** 2013-01-22

**Authors:** Giuliano Russo, Lídia Cabral, Paulo Ferrinho

**Affiliations:** 1 International Health and Biostatistics Unit, Instituto de Higiene e Medicina Tropical, Universidade Nova of Lisbon, and Centro de Malária e Outras Doenças Tropicais, Rua da Junqueira 100, Lisbon, 1349-008, Portugal; 2 Institute of Development Studies at the University of Sussex, Library road Brighton, BN1 9RE, UK

**Keywords:** Health cooperation, Aid effectiveness, Brazilian cooperation, Aid architecture, Emerging donors, Emerging economies, Brazil and Africa

## Abstract

**Background:**

Brazil is rapidly becoming an influential player in development cooperation, also thanks to its high-visibility health projects in Africa and Latin America. The 4^th^ High-level Forum on Aid Effectiveness held in Busan in late 2011 marked a change in the way development cooperation is conceptualised. The present paper explores the issue of emerging donors’ contribution to the post-Busan debate on aid effectiveness by looking at Brazil’s health cooperation projects in Portuguese-speaking Africa.

**Debate:**

We first consider Brazil’s health technical cooperation within the country’s wider cooperation programme, aiming to identify its key characteristics, claimed principles and values, and analysing how these translate into concrete projects in Portuguese-speaking African countries. Then we discuss the extent to which the Busan conference has changed the way development cooperation is conceptualised, and how Brazil’s technical cooperation health projects fit within the new framework.

**Summary:**

We conclude that, by adopting new concepts on health cooperation and challenging established paradigms - in particular on health systems and HIV/AIDS fight - the Brazilian health experience has already contributed to shape the emerging consensus on development effectiveness. However, its impact on the field is still largely unscrutinised, and its projects seem to only selectively comply with some of the shared principles agreed upon in Busan. Although Brazilian cooperation is still a model in the making, not immune from contradictions and shortcomings, it should be seen as enriching the debate on development principles, thus offering alternative solutions to advance the discourse on cooperation effectiveness in health.

## Background

Like other emerging economies, Brazil is becoming an increasingly visible development player and is contributing to the definition of new forms of cooperation among Southern nations [1]. Building on its domestic record of providing comprehensive care for HIV/AIDS [2], reforming its own health sector and moving towards universal access to its family health programme [3,4], Brazil is scaling up its engagement in health cooperation, particularly in neighbouring South American countries and Portuguese-speaking African countries [5]. Busan’s 4th High Level Forum on Aid Effectiveness, held at the end of 2011, is claimed to have changed the way development cooperation and the global aid architecture are conceptualised, particularly by broadening the debate to include non-traditional donors [6].

By looking at the evidence available on Brazil’s health cooperation projects in the five Portuguese-speaking African countries (PALOP) ^a^- Brazil’s top health development cooperation beneficiaries – the present paper aims at positioning Brazilian health cooperation within the current debate on aid effectiveness. This work draws from the evidence collected through a study funded by the World Health Organization to look at Brazilian technical cooperation and health projects in Portuguese-speaking African countries. For such study the English, Portuguese and Spanish language literature was first reviewed on Brazilian health cooperation and on aid effectiveness, and field visits were organised in the five countries and in Brazil. Seventy-sevensemi-structured interviews were conducted with key informants from government departments and implementing agencies. Project administrative and contractual documentation was reviewed for all the health projects found on the field.

Starting with an analysis of Brazil’s cooperation programme and its health projects in PALOP countries, this paper examines and discusses their adherence to the ‘development effectiveness’ principles affirmed in Busan, and tries to identify lessons to the wider debate on health sector development cooperation.

## Debate

### Brazil’s Technical health cooperation within its wider cooperation programme

Brazil’s overall cooperation programme is still relatively small, estimated to be worth between USD350 million and 1 billion per year [7,8] with a substantial component of support to international organisations and humanitarian assistance, and a smaller proportion directed to technical cooperation projects. Yet, in spite of its relatively small size in monetary terms, technical cooperation has considerable visibility and political significance, being a major instrument of Brazil’s contemporary foreign policy [9]. South-South relations play an important part in Brazil’s strategy of diversification of diplomatic and economic relations, and technical cooperation provides an expedient way of taking forward such agenda. Spearheaded by the Ministry of Foreign Affairs (Itamaraty), through its Brazilian Agency for Cooperation (ABC) [10], Brazil’s South-South technical cooperation programme has as key features the emphasis on exchange of experiences between equal partners (or ‘horizontal cooperation’, as it is usually referred to), respect for the partner country’s sovereignty, and non-conditionality of support [11]. Its technical cooperation projects have a dominant but not exclusive geographical focus on Latin American and Portuguese-speaking African countries, and on the agricultural, educational, and health sectors. Such projects typically rely exclusively on in-kind technical assistance and technological transfer, drawing on civil servants and professionals from Brazil-based institutions [12].

Because of its domestic record in fighting infectious diseases, reforming its health care system towards universal coverage, and spearheading the introduction of free Antiretroviral Treatment, Brazil has come to represent a world reference on public health [3,13,14]. Brazil has also a track record of health cooperation projects within Latin America and in those parts of the world where it can take the full advantage of sharing the Portuguese language, the most spoken in southern hemisphere and the overall 6th most common worldwide [15].

Official government figures [7] put the value of Brazilian technical health cooperation at R$24 million (approximately USD12 million), between 2006 and 2009, having evolved from a low base of R$2.8 million in 2006 to R$13.8 million (USD6 million) in 2009. However, recent independent estimates [16] valued that, for the same four-year period, Brazil spent between USD12 million and USD14 million in technical health cooperation projects in PALOP countries alone.

Brazil’s set-up for technical cooperation in health is complex and fragmented, as a multiplicity of public institutions plays key roles in either funding, management and implementation capacity [17]. Itamaraty’s ABC is supposed to play a coordinating function, officially overseeing the entire portfolio of Brazil’s technical health cooperation, but in practice it struggles to perform such function effectively [10]. Multiple health institutions are responsible for designing and implementing specific health sector projects, such as: the Brazilian Ministry of Health (MoH) and its federal advisory bodies, secretariat and departments; the Oswaldo Cruz Foundation (FIOCRUZ), a MoH-sponsored public health institution focussing on a wide array of services from research and development, to training, management of health programmes and pharmaceuticals production; and the Brazilian National Health Surveillance Agency (ANVISA). The Ministry of Education and its regulatory body, the Higher Education Coordination body (CAPES), and the Ministry of Science and Technology through its National Research Council (CNPq), also support international health cooperation activities indirectly (see Additional file 1: Table A2 on Brazilian health cooperation players).

### Emerging values and concepts

Brazil’s general as well as health sector-specific characteristics and claimed principles suggest some important departures from how development cooperation has been traditionally practiced (Table 1).

**Table 1:** Traditional and Brazilian development concepts

**Traditional aid characteristics**	**Brazil’s terminology**
Vertical donor-to-beneficiary aid	Horizontal partnership between cooperation peers
Predominantly monetary aid (grants and loans)	Predominantly in-kind technical cooperation
Focus on health programmes	Health cooperation projects ‘on demand’
Specialisation of health functions across countries according to comparative advantages	Industrial-health complex
Capacity building	Structural cooperation in health
Separation between foreign policy and development (health) goals	Health diplomacy

A key feature of Brazil’s cooperation is that it is openly driven by foreign policy goals [18], and development cooperation is seen as instrumental to promoting Brazil’s image and interests abroad. Specifically for its health cooperation, Brazil openly adopts the notion of ‘health diplomacy’ which implies that health cooperation can be informed by international health objectives, following the recognition that, in a globalised world, national health problems need to be dealt with in cooperation with international entities [19]. To this respect, some authors have taken the view that Brazil uses its health cooperation programme as a ‘soft-power tool’ to influence the global health debate, but also to promote its foreign policy interests [20,21].

Brazilian cooperation officials dispute the use of the term ‘aid’ to define their work, as that would impose industrialised countries’ “….*world views, agendas and pre-defined objectives*” [22]. Instead, ‘horizontal partnership’ is Brazil’s preferred terminology to indicate the wish to draw on principles of no-interference and mutual advantage [23]. Linked to this idea of cooperation among equals, Brazil’s preferred cooperation modality is that of technical assistance, through which government officials and technicians from beneficiary countries relate directly with their Brazilian peers, to share experiences, adopt and adapt Brazil’s technologies.

Trilateral (or triangular) cooperation is also becoming a popular recurrent feature of Brazilian development cooperation. By partnering with an international agency (often a traditional donor country or a large multilateral organisation) for a third party’s advantage (the beneficiary country), Brazilian cooperation can be scaled up and benefit from complementarities with its partner provider. The complementarity is typically between Brazil’s relevant technical expertise and the traditional donor’s or international organisation’s financial capability [24].

As for the relation between national business interests and cooperation goals, Gadelha ([2006]) puts forward the notion of Brazil’s ‘health-industrial complex for health development’, according to which individual countries need to invest in the national healthcare industry and R&D capacity if they want to develop their health systems [25]. Such emphasis on the health supply side and on self-sufficiency would also help avoiding a costly dependency on foreign healthcare technologies, as in the case of pharmaceuticals and biotechnologies [1].

Brazilian projects are also claimed to promote ‘structural cooperation in health’, a concept defined by some as building local capacity for development [26], and predicating that health cooperation should aim at: (a) integrating human resources for health and institutional development; (b) developing local capacity to avoid dependency from foreign expertise, and; (c) promoting internal collaboration between local health institutions to elaborate their own health system development agenda [27]. According to some authors [28] such views have been inspired by Brazil’s own experience of advancing health system development, which was driven by key public health institutions and social movements in Brazil that in the 1970s worked as catalyst for change.

### Brazilian health projects in PALOP countries

Portuguese-speaking Africa countries (PALOP) are the largest recipients of Brazilian health projects [29]. In 2011, there were 31 health sector-related projects in the PALOP, with Mozambique as the largest beneficiary, followed by Cape Verde and Angola (see Additional file 1: Table A3). Brazil’s MoH is the largest implementing agency, with its surveillance, AIDS and malaria departments, closely followed by FIOCRUZ. Some projects are based on triangular cooperation arrangements, carried out by Brazilian institutions but funded by other agencies, such as the Japanese International Cooperation Agency (JICA), the United Nations Population Fund (UNFPA), and the US Centers for Disease Control and Prevention (CDC) [23].

Brazil’s health projects in Africa show a pattern of established areas of support as well as emerging ones. Across the PALOP there are projects in support of individual training and training institutions, of AIDS and malaria programmes, and of strengthening the pharmaceutical area. Through CAPES/CNPq and its home universities, Brazil’s Ministry of Education offers African students training in health-related subjects such as medicine, public health and epidemiology. The offer is often limited to tuition-free openings, but at times scholarships are funded by third party international institutions. In Angola and Mozambique, FIOCRUZ directly supports locally managed national public health and health science masters. FIOCRUZ, with the support of IANPHI (International Association of National Public Health Institutes) and the CPLP (the Lusophone commonwealth), also backs the Guinean and Mozambican Institutes of Public Health and affiliated training institutions, seen as key drivers of human capital, leadership and system development in the health sector.

Capitalising on home-grown expertise and reputation, Brazil supports numerous AIDS-related projects, ranging from support to the establishment of an Antiretroviral (ARV) factory in Mozambique, to AIDS drugs donations and technical support to national AIDS programmes in Guinea Bissau and São Tomé and Príncipe. Likewise, in Angola, Cape Verde and São Tomé and Príncipe, Brazil has focussed on transferring knowledge and equipment to strengthen malaria control programmes.

Brazil’s pharmaceutical regulatory agency has been key in establishing partnerships with its counterpart agencies in Mozambique and Cape Verde to help elaborate and revise national pharmaceutical regulations. Overall, pharmaceutical sectors in Mozambique and Cape Verde benefit from Brazilian projects aimed at: (a) supporting governments’ regulatory capacity; (b) supporting public pharmacies; (c) creating local manufacturing capacity, and; (d) donating pharmaceuticals. Human Milk Banks and dental care represent other areas of emerging cooperation in these two countries, capitalising on successful experiences back home [30].

### The new ‘aid effectiveness’ equilibrium from Busan: business as usual with additional players?

How does Brazil’s experience of cooperation in health, relate to more established practices of traditional donors, and how does it reflect or contribute to current debates on ‘aid effectiveness’?

In the health sector – seen by many as providing an insight on other development sectors because of its relevance and complexity - recent evidence have shown that a large proportion of development funds are still channelled through projects, remains unpredictable, and pressure to demonstrate results in the short-term reduces opportunities for long-term reforms, which distorts health priorities, increases transaction costs and undermines national systems [31]. A number of initiatives (such as the International Health Partnership+) have been therefore launched drawing the attention on the importance of better monitoring donors’ as well as country partners’ commitments to aid effectiveness standards in the health sector [32]. However, the increasingly noticeable presence of emerging cooperation actors in the developing world, carrying new approaches to cooperation as well as using different modalities – has rapidly exposed the limitations of the prevalent aid paradigm.

In late 2011 the international development community convened in Busan, South Korea, to discuss and agree on principles on how to deliver effective assistance to developing countries [33]. One particular novelty was the sheer representation of non-traditional donors and so-called South-South cooperation providers, as for the first time, countries like China, India and Brazil sat alongside traditional donors to establish common principles to guide their practices as providers of development cooperation.

Busan introduces important shifts in the ‘aid effectiveness’ framework, whose contours had been defined at the second High Level Forum on Aid Effectiveness, held in 2005 in Paris, and reaffirmed at the Accra Forum in 2008 [34]. Above all, Busan makes space for a more inclusive partnership for international development, not only by highlighting the role of South-South cooperation and non-governmental actors, but also by acknowledging the importance of non-aid forms of development finance and proposing changes to the governance of the global aid system.

**Figure 1:**
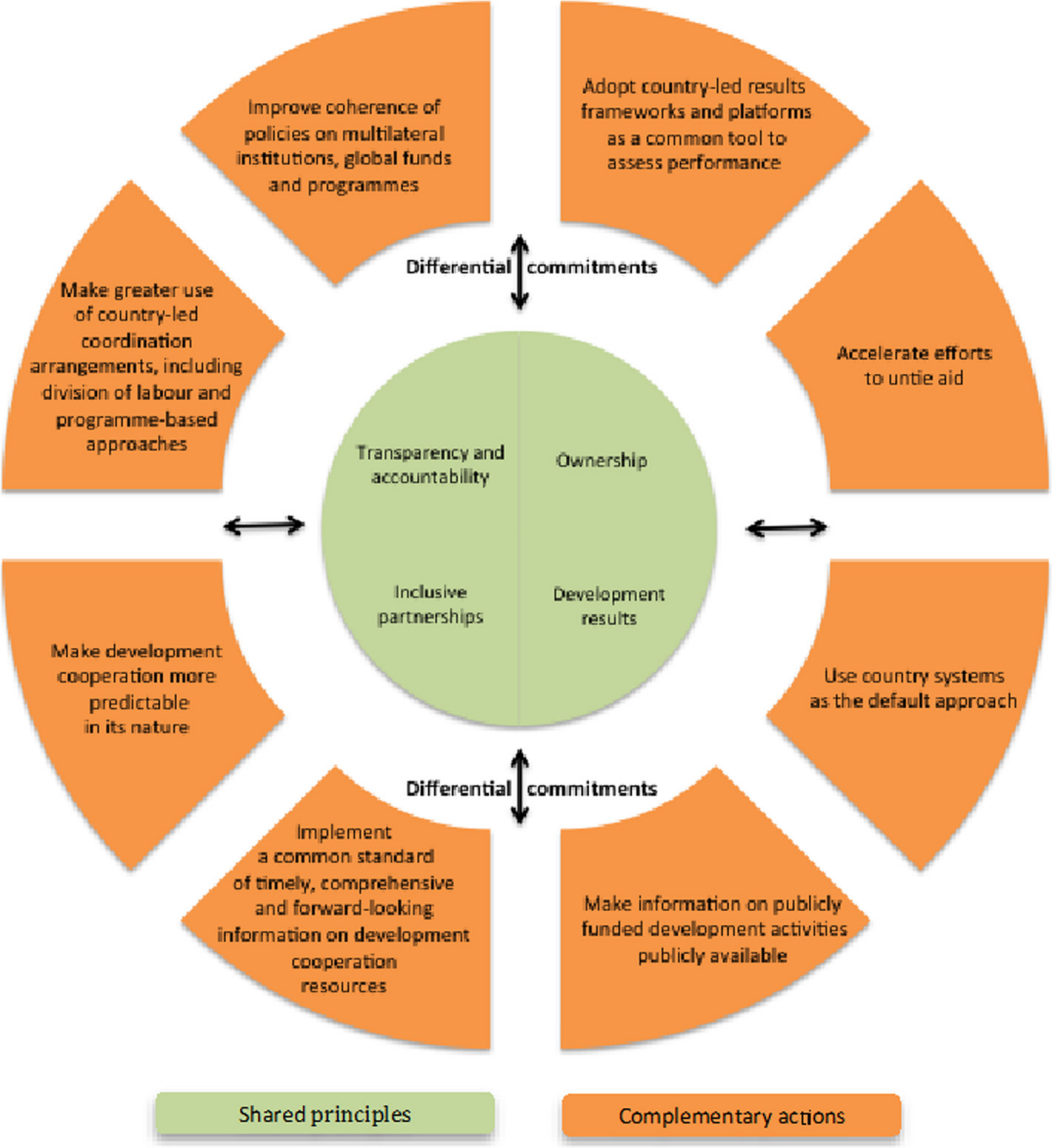
Key concepts from Busan’s aid effectiveness– shared principles, complementary actions, differential commitments.

The agreed set of shared principles reflects the latest debates on development effectiveness and reaffirms some of the commitments made in previous High Level *Fora* (Figure 1). Such principles include:

(i) *Country-ownership* - meaning that developing countries have to lead the process of setting development priorities for the partnerships for development to be successful;

(ii) *Development results* - keeping focused on the lasting impact of investments and efforts in reducing poverty, inequality and sustainable development, as well as improving developing countries capacities to tacke these issues;

(iii) *Inclusive partnerships* – establishing partnerships which have openness, trust and mutual respect and learning at their core, thereby acknowledging the complementary role of all actors in the partnership; and

(iv) *Transparency and accountability* – ensuring that this applies not only within the partnership but also in relation to the intended beneficiaries of development processes, such as the citizens, constituents and other shareholders from the countries providing as well as receiving cooperation.

Busan also established a number of complementary actions – on untying aid, predictability of development cooperation and use of country systems, among others (see Figure 1 for a selection) – applicable differently across members of the platform. The ‘differential commitments’ provision is an important mark of the Busan outcome document, reflecting the need for compromise on less consensual issues to sustain the broader partnership. It leaves behind, however, a great deal of ambiguity regarding the applicability of the individual complementary actions (the meat of the agreement) across the different membership categories [35].

Although Busan has gone a long way towards building up a common platform for traditional and emerging donors, some consider it has failed to define commitments, particularly for the platform’s new members, delegating to a subsequent technical level the mundane issue of setting time-bound and measurable targets [36]. Especially for the health sector, Busan’s ramifications are still an unknown quantity, and some fear that the absence of tangible pledges and the delegation of sector specific commitments to country level processes threat to compromise progress on aid effectiveness in health [37].

### Brazil’s Health cooperation projects in Africa from a ‘development effectiveness’ perspective

The more inclusive development cooperation framework put forward in Busan seems a more realistic way of making sense of the quickly evolving architecture of international development. The emphasis on differential paths and complementarities is more attuned to the variety of approaches to development cooperation in operation. And the focus on development effectiveness, rather than on aid effectiveness, is a more straightforward account of the blurred divide between solidarity, business and diplomacy as drivers of cooperation. Brazil’s particular experiences of supporting health systems in developing countries seem to fit well into this new framework. Concepts such as ‘health diplomacy’ and ‘health-industrial complex’ not only reflect the overlaps between the different drivers of cooperation but also contribute to give it a legitimate justification to domestic constituencies. Furthermore, Brazil’s particularly bold stance on certain health issues, such as on HIV/AIDS fight, shows the value of having differential paths toward addressing development issues.

Yet, adherence to the specific commitments set in Busan for all development players has been somewhat mixed. The absence of policy conditionalities and the emphasis on capacity building for development contribute to the efforts at enhancing **country ownership**, although Brazil’s exclusive recognition of national governments as cooperation counterparts may at times be seen as a limitation if the beneficiary national governments do not have the capacity to meet the partnership criteria. However, agreeing cooperation projects only if expressly solicited by national government is also seen as hampering the development of more consistent and comprehensive health cooperation country programmes.

Existing Government’s cooperation publications and project reports tend to focus more on administrative issues than on project results. The fragmentation of Brazilian cooperation projects, their focus on short-term action, and the absence of clearly formulated sector programmes, are all factors making it difficult to evaluate project results. The absence of a unified system to monitor and evaluate health cooperation projects may be signalling Brazil’s lack of focus on **results for sustainable development**.

It would appear that Brazil has been able to provide leadership in the countries with whom it cooperates without exerting a dominant role, arguably because of: the absence of monetary transfers in its cooperation projects; the exclusively ‘reactive’ nature of its cooperation to local governments’ requests; the absence of policy conditionalities, and; its reliance on domestic civil servants rather than on aid professionals. Brazilian cooperation officials are credited with creating a highly praised **rapport with local counterparts**, in part explained by the common language and by shared cultural roots with those countries. As Brazilian cooperation officials are civil servants performing similar duties back home, local counterparts tend to consider them more as ‘foreign colleagues’. However, such lack of development-specific expertise is also reported to have its downsides, especially in terms of Brazilians’ ability to capture the complexities of project implementation in low-income settings.

The notions of **accountability and transparency** do not appear often in Brazil’s cooperation documents. Regular publications exist on Brazilian health projects in Africa [15], but these usually fall short of meeting international standards on transparency and publicity of information, especially on its financial aspects. At the local level, there is no system to track information on specific projects, and Brazilian Embassy offices at country level often lack basic information on those projects they do not manage directly. With the notable exception of triangular cooperation projects, peer review of Brazilian health cooperation is almost inexistent, as Brazil seldom participates in local health *fora*.

### Summary

The new equilibrium set in Busan gives a greater role to non-traditional development cooperation players. Together with other emerging donors, Brazil is credited to have already contributed to move forward the debate from aid to development cooperation and an argument could be made that Busan represents a synthesis, albeit a blurred one, of old and new development paradigms.

Brazilian health cooperation is contributing to advance the debate on international development by adopting new concepts such as ‘health diplomacy’, ‘health-industrial complex’ and ‘structural cooperation’. Brazilian support to health development abroad also challenges conventional wisdom on the HIV-AIDS fight, on the separation between foreign policy and health objectives, and on the role played by the national health industry. Brazilian cooperation officials also bring a refreshing attitude to cooperation, based on a practical approach to cooperation and tested solutions, but also on a rapport with their counterparts that openly brings trade and foreign policy issues into the cooperation equation. A key feature of their approach is the careful and consistent usage of a language that emphasizes their key principles even when concrete action-content is missing. This rhetoric is essential to present and reinforce a public image of “concerned and responsive partner”.

However, Brazilian cooperation is still a model in the making, and inconsistencies are starting to appear as its programme grows and is confronted with the complexities of projects’ impact and sustainability in resources-poor African settings. Tangible results from Brazilian health cooperation projects’ are still to be demonstrated, partly because they are too recent to have produced an impact, but also because of Brazilian government’s apparent lack of interest in evaluating its cooperation programme. At a time when increased attention is given to project results and health impact [31,32] Brazil appears conspicuously out of step on this.

Brazilian health projects claim to be inspired by newly affirmed principles but, with the notable exception of the support to the ARV factory in Maputo, it is sometimes difficult to see behind the rhetoric to what extent Brazilian health projects are truly ground-breaking and differ from traditional capacity building and technical assistance. Finally, our analysis has shown how Brazilian health projects only selectively comply with the shared cooperation principles agreed upon in Busan; failing to show progress on these could compromise Brazil’s claim of adding value to the debate.

Brazil’s main merit so far is still one of bringing salutary diversity to the debate on how health development cooperation should be framed and on the modalities of its delivery, which has been credited with advancing development science [38] and offering recipient countries a richer array of solutions to choose from, together with a vital negotiating space with dominant development institutions [39,40]. As some of its largest health projects appear to show, Brazil is already a presence to be reckoned with in the health arena. The message from Busan may be that engaging with new players and exploring synergies between traditional and new forms of development cooperation, appears to be the only way forward.

## Endnote

^a^ Angola, Cape Verde, Guinea Bissau, Mozambique and São Tomé and Príncipe. Source: Authors’ own elaboration.

## Competing interests

The authors declare that they have no competing interests.

## Authors’ contributions

GR carried out data collection and drafted the original manuscript. LC and PF participated in drafting the manuscript and edited the final version. All authors read and approved the final manuscript.

## Authors’ information

GR is a research fellow and lecturer in health economics at the Institute of Hygiene and Tropical Medicine of Lisbon, a WHO Collaborating Centre on Human Resources for Health. GR has an extensive academic and research experience in Africa and Latin America, and his recent research has focussed on health systems in low-income countries, aid architecture and project evaluation, with a specific regional interest on the Community of Portuguese-Speaking Countries.

LC is a research fellow associate of the Overseas Development Institute, and a PhD candidate at the Institute of Development Studies of Brighton. An agriculture economist and public finance specialist with extensive professional experience in Africa and Brazil, LC has published on aid effectiveness, and has been more recently involved in organising events on the Brazilian development cooperation.

Paulo Ferrinho is director of the Institute of Hygiene and Tropical Medicine of Lisbon. Medical doctor, epidemiologist and public health specialist, PF has published extensively on human resources for health in Europe and Africa and health systems research with a specific focus on Lusophone Africa. PF is currently managing a collaboration with Brazilian public health institutions aimed at strengthening organisation and health policies of the Community of Portuguese- speaking Countries.

## Supplementary Material

Additional file 1: Table A2 Brazilian governmental institutions directly involved in international health cooperation. **Table A3.** Brazilian health projects found on the field in Portuguese-speaking African countries in 2011. Click here for file

## References

[B1] GHSi: Shifting paradigma How the BRICS are reshaping global health and development 2012 Global Health Initiatives Organization Accessed on 29/03/2012 at: http://www.ghsinitiatives.org/downloads/ghsi_brics_report.pdf

[B2] Le Loup G de Assis A Costa-Couto MH A public policy approach to local models of HIV/AIDS control in Brazil Am J Public Health 2009 99 1 8 10.2105/AJPH.2008.138123 PMC2679776 19372523

[B3] Paim J Travassos C Almeida C Bahia L Macinko J The Brazilian health system: history, advances and challenges Lancet 2011 377 1778 1797 10.1016/S0140-6736(11)60054-8 21561655

[B4] WHO Bulletin Brazil’s march towards universal coverage Bull World Health Organ News 2012 88 646 647 doi:10.2471/BLT.10.020910 10.2471/BLT.10.020910 PMC2930369 20865066

[B5] Buss PM Health diplomacy and South-South cooperation: the experiences of UNASUR Salud and CPLP¹s Strategic Plan for Cooperation In Health Eletr Rev of Com Inf Innov and Health 2010 14 1 98 110

[B6] Hill P Brown S Haffeld J Effective aid in a complex environment Bull World Health Organ 2011 89 854 854A 10.2471%2FBLT.11.098285 22271936 10.2471/BLT.11.098285 PMC3260903

[B7] IPEA Cooperação Brasileira para o Desenvolvimento Internacional 2005–2009 Casa Civil, IPEA e ABC 2011 2011 Brasília: Administração pública federal

[B8] The Economist Speak softly and carrying a blank cheque 2011 The Economist 2010 Accessed on 07/2011 at: http://www.economist.com/node/16592455

[B9] Vigevani T Cepaluni G Lula’s foreign policy and the quest for autonomy through diversification Third World Quarterly 2007 28 7 1309 1326 10.1080/01436590701547095

[B10] Cabral L Weinstock J Brazil: an emerging aid player - Lessons on emerging donors, and South-South and trilateral cooperation ODI Briefing Paper 2010 London: Overseas Development Institute 64

[B11] ECOSOC Background study for the Development Cooperation Forum. Trends in South-South triangular development cooperation 2008 Geneva: United Nations Economic and Social Council

[B12] AISA Relatório de Gestão 2009 da Assessoria de Assuntos Internacionais de Saúde 2010 Brasília – DF: Ministério da Saúde 2010

[B13] Barreto ML Teixeira MG Bastos FI Ximenes RAA Barata RB Rodrigues LC Successes and failures in the control of infectious diseases in Brazil: social and environmental context, policies, interventions, and research needs Lancet 2011 377 9780 1877 1889 10.1016/S0140-6736(11)60202-X 21561657

[B14] Victora CG Aquino EML Leal MC Monteiro CA Barros FC Szwarcwald CL Maternal and child health in Brazil: progress and challenges Lancet 2011 377 9780 1863 1866 10.1016/S0140-6736(11)60138-4 21561656

[B15] Lewis MP Ethnologue: Languages of the World 2009 16 Dallas: Tex.: SIL International Accessed on 25/05/2011 at: http://www.ethnologue.com/print.asp

[B16] Russo G Ferrinho P Dussault G Floriano A Brazilian cooperation’s health projects in Portuguese-speaking African countries Instituto de Higiene e Medicina Tropical 2011 2011 Lisbon: WHO study report

[B17] Bliss KE Health in All Policies; Brazil’s Approach to Global Health within Foreign Policy and Health Cooperation Initiatives. In: “Key Players in Global Health: How Brazil, China, India, Russia and South Africa are influencing the Game 2010 Washington: CSIS Global Policy Center

[B18] Saraiva MG Brazilian foreign policy towards South America during the Lula administration: caught between South America and Mercosur Rev. Bras. Polít. Volume 53 2010 Brasília: Int 2010

[B19] Kickbusch I Silberschmidt G Buss P Global health diplomacy: the need for new perspectives, strategic approaches and skills in global health Bull of the World Health Org 2007 85 3 230 232 10.2471/BLT.06.039222 PMC2636243 17486216

[B20] Feldbaum H Michaud J Health Diplomacy and the Enduring Relevance of Foreign Policy Interests PLoS Med 2010 7 4 e1000226 10.1371/journal.pmed.1000226 20422036 PMC2857879

[B21] Lee K Chagas LC Novotny TE Brazil and the Framework Convention on Tobacco Control: Global Health Diplomacy as Soft Power PLoS Med 2010 7 4 e1000232 10.1371/journal.pmed.1000232 20421917 PMC2857639

[B22] Buss PM Ferreira JR Critical essay on international cooperation in health RECIIS –Eletr Rev of Com Inf Innov Health 2010 4 1 86 97

[B23] ABC Cooperação técnica brasileira em saúde Via ABC 2007. Boletim eletrônico da Agência Brasileira de Cooperação (ABC/MRE) 2007 Março 24 Available at: http://www.abc.gov.br/intranet/Sistemas_ABC/siteabc/documentos/viaABC-baixa.pdf (Access on 01/August/2009)

[B24] CIDA Programa Brasil-Canadá para a promoção da equidade. PIPE – cooperação trilateral, folheto 7 2007 Gatineau: Agência Canadense para o Desenvolvimento Internacional CIDA

[B25] Gadelha CAG Desenvolvimento, complexo industrial da saúde e política industrial Rev Saude Publica 2006 40 11 23 N Esp 10.1590/S0034-89102006000400003 16924298

[B26] Almeida C Pires De Campo R Buss P Ferreira JR Fonseca LE Brazil’s conception of South-South “structural cooperation” in health RECIIS –Eletr Rev of Com Inf Innov and Health 2011 4 1 23 32

[B27] Buss PM Brazil: structuring cooperation for health Lancet 2011 377 9779 1722 1723 10.1016/S0140-6736(11)60354-1 21561654

[B28] Fleury S Brazil’s health care reform: social movements and civil society Lancet 2011 377 9779 1724 1725 10.1016/S0140-6736(11)60318-8 21561650

[B29] MoH of Brazil Relatório de gestão 2009 da Assessoria de Assuntos Internacionais de Saúde 2011 Brasília: MS-AISA

[B30] Aia PRS Novak FR Almeida JRG Silva DA The management strategy of the Brazilian National Network of Human Milk Banks Cad Saude Publica 2004 20 6 1700 1708 10.1590/S0102-311X2004000600029 15608873

[B31] OECD Aid effectiveness in the health sector: progress and lessons 2012 Better Aid, OECD Publishing http://www.oecd-ilibrary.org/development/progress-and-challenges-in-aid-effectiveness_9789264178014-en

[B32] Sorten T Taylor M Spicer N Mounier-Jack S McCoy D The International Health Partnership Plus: rhetoric or real change? Results of a self-reported survey in the context of the 4th high level forum on aid effectiveness in Busan Glob Heal 2012 8 13 10.1186/1744-8603-8-13 PMC3519754 22650766

[B33] HLF4 Busan Partnership for Effective Development Co-operation 2011 Busan, South Korea: Fourth High Level Forum on Aid Effectiveness

[B34] Martini J Mongo R Kalambay H Fromont A Ribesse N Dujardin B Aid effectiveness form Rome to Busan: some progress but lacking bottom-up approaches or behaviour change International Medicine and Tropical Health 2012 17 7 931 933 10.1111/j.1365-3156.2012.02995.x 22583911

[B35] Cabral L Russo G Weinstock J **Brazil and the shifting consensus on development cooperation. Salutary diversions from the ‘aid effectiveness’ trail.** Dev Pol Rev In press

[B36] Glennie J Busan has been an expression of shifting geopolitical realities 2011 The Guardian, 2nd December: Poverty Matters Blog

[B37] Golichenko O Global health cooperation: what is next? The Broker – connecting world of knowledge blog 2012 Accessed on 27/02/2012 at: http://www.thebrokeronline.eu/Blogs/Busan-High-Level-Forum/Global-health-cooperation-what-is-next

[B38] Adésinà JO Social Policy in Sub-Saharan African Context: In Search of Inclusive Development National research Institute for Social Development 2007 Palgrave, Houndsmills: National research Institute for Social Development

[B39] King K New Actors – Old Paradigms? NORRAG News 2010 44 8 12

[B40] Kragelund P The Return of Non-DAC Donors to Africa: New Prospects for African Development? Dev Pol Rev 2008 26 5 555 584 10.1111/j.1467-7679.2008.00423.x

