# Supplementary material for: Brazil-Africa technical cooperation in health: what’s its relevance to the post-Busan debate on ‘aid effectiveness’?
Source: Global Health. 2013 Jan 22;9:2. doi: 10.1186/1744-8603-9-2 (PMC3610229; doi:10.1186/1744-8603-9-2)
Supplement: Additional file 1: Table A2 — Brazilian governmental institutions directly involved in international health cooperation. Table A3. Brazilian health projects found on the field in Portuguese-speaking African countries in 2011. [file 1744-8603-9-2-S1.doc]

# Annex II: Brazilian Cooperation health projects in Portuguese-speaking African Countries found on the field in 2011

| **Country** | **Project** | **Implementing Agency** | **Status** |
| --- | --- | --- | --- |
| Angola | Health System Capacity Building: Masters in Public Health ^1^ | FIOCRUZ/MoH | Ongoing |
|  | Pilot Project on Sickle-cell Disease ^1^ | MoH | In negotiation |
|  | Support to the malaria prevention and control programme ^1^ | SVS/MoH | Phasing out |
|  | Technical support to development of the Angola Military Centre for Hygiene and Epidemiology ^1^ | Minas Gerais Federal University | In negotiation |
|  | Triangulation Project between JICA-Brazil-Angola to build capacity at the Josina Machel Hospital ^1^ | Campinas and Sao Paulo Universities, Santa Cruz Hospital, Sofia Feldman Hospital/JICA | Phasing out |
| Cape Verde | Primary care system strengthening in Cape Verde ^2^ | NATES/UFJF (Minas Gerais) | Ongoing |
|  | Strengthening of the national food and drug regulatory agency ^2^ | ANVISA | Ongoing |
|  | Support to the national malaria control and prevention ^2^ | SVS/MoH | Ongoing |
|  | Technical support for the implementation of Human Milk Banks in Cape Verde ^2^ | FIOCRUZ/MoH | Ongoing |
| Guinea Bissau | Building local capacity to respond to gender-based violence ^3^ | MoH | Ongoing |
|  | Support to the AIDS fight programme and ARV donations ^3^ | SVS/MoH | Ongoing |
|  | Support to the INASA Public Health Laboratory ^3^ | Rio de Janeiro Federal University | In negotiation |
|  | Support to the National AIDS Secretariat ^3^ | SVS/MoH | In negotiation |
|  | Support to the national Health Institute (INASA) ^3^ | FIOCRUZ/MoH | Ongoing |
| Mozambique | ARV Factory ^4^ | FIOCRUZ/MoH | Ongoing |
|  | Breast and uterine cancer prevention ^4^ | INCA/ MoH | Signed |
|  | Dental health – setting up a laboratory for dental prostheses ^4^ | MoH | Signed |
|  | Epidemiological study in dental health ^4^ | MoH | Signed |
|  | Human milk bank project ^4^ | FIOCRUZ/ MoH | In negotiation |
|  | INS strengthening ^4^ | FIOCRUZ/ MoH | Ongoing |
|  | Master programme in health sciences ^4^ | FIOCRUZ/ MoH | Ongoing |
|  | Mental health programme ^4^ | University of Brasília NGO | Ongoing |
|  | Mother, child and adolescent health ^4^ | MoH | In negotiation |
|  | Strengthening the pharmaceutical department’s regulatory agency ^4^ | ANVISA | Ongoing |
|  | Support to FARMAC ^4^ | FIOCRUZ/ MoH | Ongoing |
|  | Trilateral cooperation in HIV/AIDS ^4^ | FIOCRUZ/MoH CDC, USAID | In negotiation |
|  | Upgrading the CRDS into mid-level health personnel training polytechnic ^4^ | FIOCRUZ/ MoH | In negotiation |
| São Tomé and Príncipe | Support to Logistics, Supply Systems and Epidemiologic Surveillance (TB, HIV/AIDS and Syphilis) System ^5^ | SVS/MoH | In negotiation |
|  | Support to the HIV/AIDS prevention and control programme ^5^ | SVS/MoH | Ongoing |
|  | Support to the Malaria Prevention and Control programme ^5^ | SVS/MoH | Ongoing |
|  | Support to the Tuberculosis Control Programme ^5^ | SVS/MoH | Ongoing |

*Sources: Brazilian Embassies in Angola (1), Cape Verde (2), Guinea Bissau (3), Mozambique (4) and São Tomé and Príncipe (5)*
